# Understanding the Effect of Surface Machining on the YSZ/Ti6Al4V Joint via Image Based Modelling

**DOI:** 10.1038/s41598-019-48547-w

**Published:** 2019-08-19

**Authors:** Chun Li, Xiaoqing Si, Xiangyu Dai, Xun Zhang, Ying Chen, Junlei Qi, Zhibo Dong, Jicai Feng, Jian Cao

**Affiliations:** 1 State Key Laboratory of Advanced Welding and Joining, Harbin Institute of Technology, Harbin, 150001 China; 2 School of Materials, University of Manchester, Oxford Road, Manchester, M13 9PL United Kingdom; 3 OxMet Technologies, Begbroke Science Park, Oxfordshire, OX5 1PF United Kingdom

**Keywords:** Design, synthesis and processing, Ceramics

## Abstract

A method to improve the brazing between YSZ and Ti6Al4V by femtosecond laser surface machining is introduced. The highest strength of ~150 MPa (which is 95.2% higher than that of the flat YSZ/Ti6Al4V joint) is achieved when the processing speed is 200 μm/s. To understand the strengthen mechanism of the surface machining on the joint strength, image based models, based on the observed microstructure, have been used to probe the stress distribution in the joint. It is found that through surface machining on the ceramic, the residual stress distribution in ceramic becomes nonlinear. Upon shear testing, for the joint with a flat interface, the failure happens in the reaction layer and the out of plane stress in this layer is found to be tensile, which acts as the driving force for the crack generation and propagation. But for the joint with a rumpled interface, the compressive out of plane stress at the boundary of the grooves in the reaction layer could inhibit the propagation of the cracks. Finally, by surface machining on the ceramic, the maximum shear stress in the reaction layer is decreased, which could also help to improve the reliability of the joint.

## Introduction

Yttria stabilised zirconia (YSZ) is a ceramic material widely used as thermal barrier coatings^1^ and electrolyte layers^2,3^. In these applications, the ceramic is often bonded with metals to realise its functional or mechanical applications. Active brazing^4,5^ is a practical method to join ceramics or join ceramics to metals, in which reactive elements such as Ti, Zr and Hf are incorporated into the brazing alloys to modify the chemistry of the ceramic surface and to improve the wettability and adhesion of the brazing alloy on ceramics. Numerous research^5–8^ has been carried out to investigate the active brazing between ceramics and metals and two main challenges are often encountered. The first one is the control of the reaction between the brazing alloy and the ceramics to avoid the excessive formation of brittle reaction products at the interface. This problem can often be properly solved by modifying the composition of the brazing alloy and using the optimised brazing parameters^9,10^. The other challenge is the residual stress generated from the mismatch of the coefficient of thermal expansion (CTE) between the ceramic and the metal. The residual stress is detrimental to the reliability of the joint and sometimes can directly lead to the failure of the joint. A considerable amount of literature has been published on strategies to relax the residual stress in ceramic/metal brazing joint. Three most commonly used methods are: (i) by applying a soft and ductile interlayer such as copper foil^11^, foam^12^ and niobium foil^13^ which could release the residual stress in the joint via plastic deformation. This method is considered to be of limited success because the soft interlayer usually has relatively low melting temperature and the addition of the interlayer will inevitably be detrimental to the high temperature mechanical properties of the joint^14^. (ii) By applying an interlayer with a CTE value between the two substrates, which could form a gradient structure in the joint and moderate the residual stress distribution^15^ and (iii) by incorporating secondary phases which usually have a low CTE^16,17^ into the brazing alloy to tailor the CTE of the brazing alloy. For the last two methods, the additional interlayer or secondary phases must be compatible in both CTE and chemical composition with the material system in the brazing joint. However, sometimes it can be quite challenging to find proper candidates. Thus it is desirable to develop a novel method to optimise the residual stress distribution in ceramic/metal brazing joint.

Residual stress is the driving force for the failure of the ceramic coatings^1,18^. Improved mechanical keying through a rough interface profile is widely used in ceramic/metal systems to increase the interface adhesion and the lifetimes of the components^19^. It was found that this undulate interface could have a considerable influence on the residual stress distribution across the coating^20^. Thus it is expected that a well-designed rumpled interface for a brazing joint may also be able to optimise the residual stress distribution and benefit the bonding quality. Some research has been carried out to modify the surface morphology of ceramic prior to brazing. Xiong *et al*.^21^ machined a series of rectangular grooves on the surface of C/C composite material and then brazed it with Ti6Al4V alloy. It was predicted by FE (Finite Element) models that the residual stress distribution in the joint was modified and the shear strength of the joint was found to be increased. Shen *et al*.^22^ and Hernandez *et al*.^23^ machined the surface of C/C and C_f_/SiC composite materials by hole drilling before brazing and achieved strengthened joint with Ni-based superalloy and titanium alloy. However, the depth of the patterns machined usually ranges from several hundred microns to several millimetres, which might be detrimental to the mechanical properties of the substrates. Femtosecond laser has become one of the most promising methods for patterning the surface of ceramics^24–26^ and metals^27–29^ because it could create relatively shallow grooves with little thermal damage. Yang *et al*.^30^ carried out surface machining on alumina surface via femtosecond laser and the achieved grooves were less than 100 μm deep. The strength of the joint with stainless steel is about 2.7 times higher than that with a flat interface. The effect of the laser power on the joint strength was investigated and the optimised laser power was found to be 0.3 W. Unfortunately, the effect of the machining speed which is an important parameter for laser machining^31^ on the microstructure and mechanical property of the joint was not discussed. The authors also predict the residual stress distribution along the interface by FE models and found it was altered by the surface machining. This result, however, has failed to present the residual stress distribution in the ceramic substrate near the interface and the reaction layer adjacent to the ceramic where the actual failure usually happens. And up to now, no research is available on the effect of surface machining of YSZ on the residual stress distribution in the interface area.

Residual stress has a pivotal role in the reliability of the brazing joint and a considerable number of research has been carried out to model its distribution in the joint. Iancu *et al*.^32^ developed an analytical model to calculate the residual stress distribution as a function of depth in the brazing joint. It was predicted when the CTE of ceramic is smaller than that of the metal, the residual stress is compressive in the ceramic and increases from the surface to the interface. FE models have also been utilised to investigate the residual stress distribution in brazing joints between ceramic and metals such as Si_3_N_4_/Invar^33^, BN/AISI 1045 steel^34^, alumina/Kovar^35^ and zirconia/cast iron^36^. The results generally show that the residual stress in ceramic is compressive. However, all the previous research has only been restricted to using artificial or simplified models which regard the brazing seam as a homogeneous layer and all the interfaces are flat. In reality, brazing joints usually have a complex microstructure which consists of various phases with undulated morphology^4,5^, which could have great influence on the residual stress distribution. Unfortunately, no previous study has taken the morphology of the phases into consideration when investigating the residual stress distribution in the joint.

Image based modelling is a method which incorporates real microstructure of material into FE models. This method has been successfully applied to investigate the residual stress distribution in materials with relatively complex microstructure such as thermal barrier coatings (TBC)^37^ and porous materials^38,39^. Therefore, it is expected that the residual stress distribution in a brazing joint could be modelled using a similar approach.

In this study, a new method to modify the residual stress distribution in a YSZ/Ti6Al4V alloy brazing joint and to improve the joining quality via femtosecond laser surface machining is introduced. The effect of the machined surface morphology on the microstructure and the mechanical properties of the joint are studied. The effect of the surface machining on the residual stress distribution and stress distribution during shear testing in the brazing joint is investigated by image based models based on the observed microstructure of the joint.

## Results and Discussion

### The microstructure of the joint

**Figure 1:**
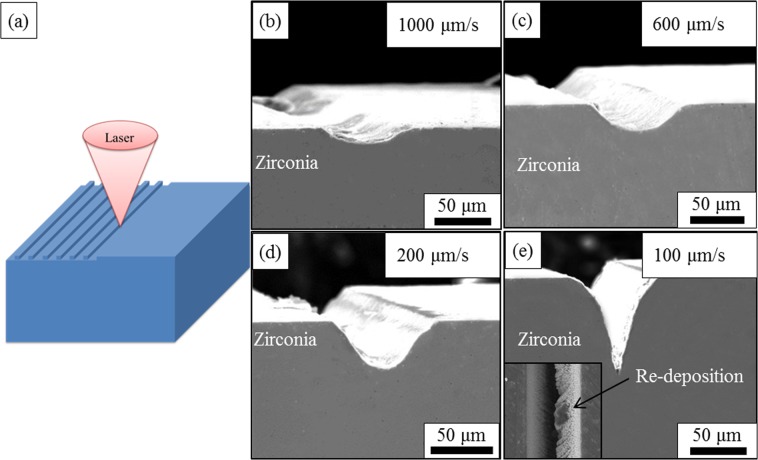
The morphology of the zirconia surface after surface machining by femtosecond laser with different processing speed, showing that with the decreasing of the processing speed, the depth of the machined grooves increase. (**a**) The schematic showing the surface machining process, and the morphology of the zirconia surface after femtosecond laser surface machining with a processing speed of (**b**) 1000 μm/s, (**c**) 600 μm/s, (**d**) 200 μm/s, (**e**) 100 μm/s, a sharp corner can be observed at the concave of the machined grooves.

The surface morphologies of YSZ after machining by femtosecond laser with different processing speeds are shown in Fig. 1.

From Fig. 1(b–e), it can be seen that grooves can be successfully machined on the surface of the YSZ when the pulse energy was 200 μJ, the repetition rate was 1 kHz and the grooves are relatively smooth. The processing speed seems to have no large influence on the width of the grooves, which is around 60 μm for all the samples when the processing speed ranges from 100 μm/s to 1000 μm/s, as shown in Fig. 1. However, the processing speed has a great impact on the depth of the grooves. From Fig. 1 we can see that as the processing speed rises from 100 μm/s to 1000 μm/s, the depth of the grooves drops from ~63 μm to ~15 μm. The slower processing speed could lead to a higher energy input to the ceramic and result in the increase of the processed grooves. It needs to be noted that when the processing speed is higher than 100 μm/s, the bottom of the groove is relatively smooth. However, when the processing speed is lowered to 100 μm/s, a sharp corner appears at the bottom of the groove and re-deposition can be observed (shown in the insert of Fig. 1(e)). This could be attributed to the high energy input induced by the low processing speed which evaporates the materials and some of the vapour cannot escape from the groove, forming the deposition^40^.

**Figure 2:**
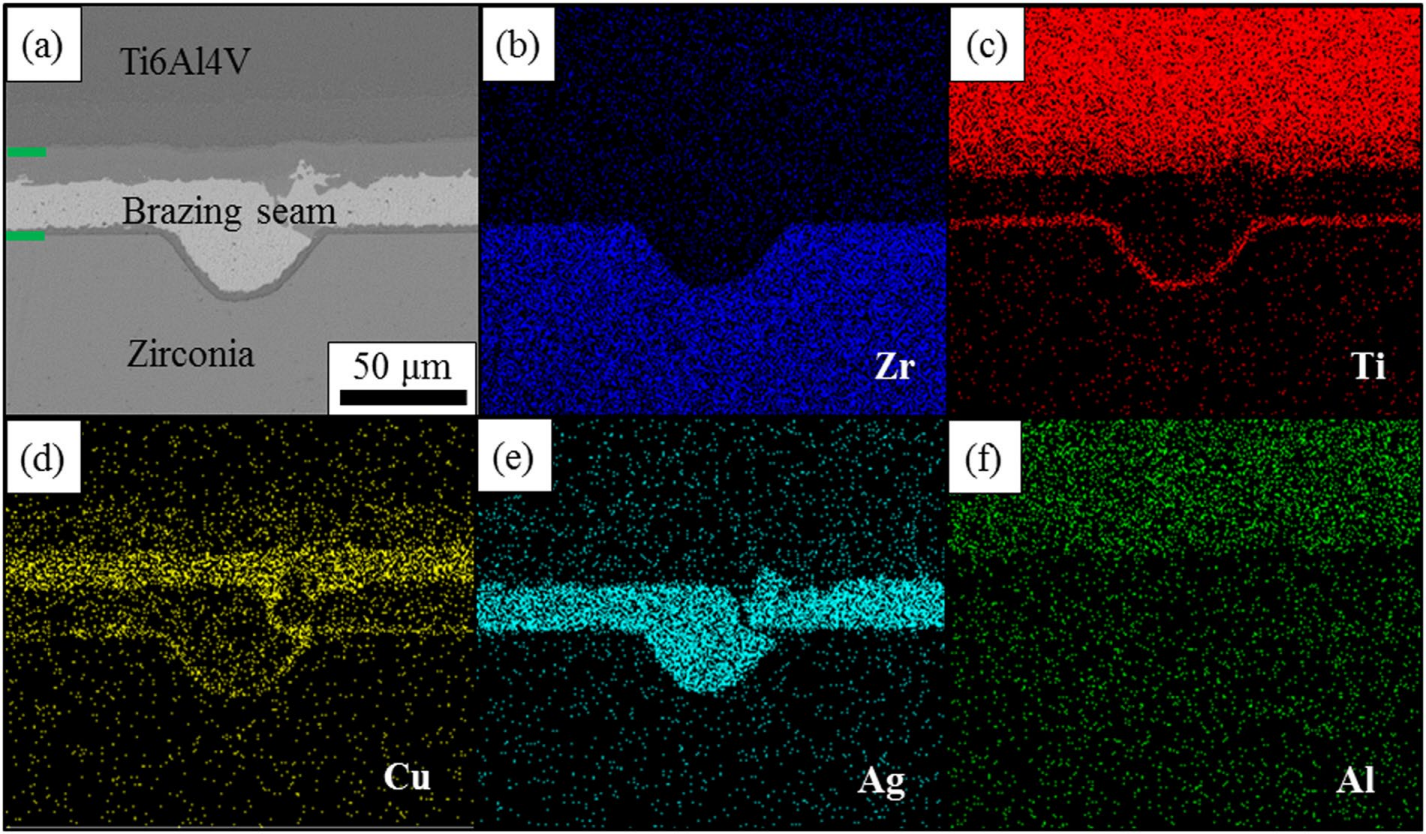
The typical microstructure of surface machined zirconia/Ti6Al4V joint and the distribution of various elements in the joint, (**a**) the microstructure of the surface machined zirconia/Ti6Al4V joint brazed by AgCu brazing filler at 850 °C for 10 min which demonstrates that brazing filler can successfully fill up the grooves induced by surface machining, (**b**) the distribution of Zr in the joint which mainly locates inside zirconia, (**c**) the distribution of Ti in the joint, which can be found both inside Ti6Al4V alloy and the reaction layers, (**d**) the distribution of Cu in the joint and it is mainly concentrated in the reaction layers, (**e**) the distribution of Ag in the joint which locates inside the silver solid solution and (**f**) the distribution of Al in the joint which can only be found inside the Ti6Al4V alloy.

The typical microstructure of the surface machined YSZ/Ti6Al4V joint brazed at 870 °C for 10 min and the corresponding element maps are shown in Fig. 2. As shown in Fig. 2(a), the joining between the surface machined YSZ and Ti6Al4V alloy was successfully achieved without any defects such as cracks and pores. The brazing filler was found to fill up the grooves fabricated by femtosecond laser and continuous reaction layers could be observed at the interface between the ceramic and the brazing filler along both the grooves and the flat parts. This indicates that the surface machining process has no adverse effect on the reaction between the ceramic and the brazing filler. Figure 2(b–f) show the distribution of the various elements in the joint. Ti and Cu were found to segregate at the interface between YSZ and the brazing filler, from which it can be inferred that after the melting of the brazing filler, Ti from Ti6Al4V diffused into the brazing seam and reacted with YSZ. Al and Zr only distribute inside Ti6Al4V and YSZ while Ag distributes in the middle of the brazing seam. From this figure, we can see that despite the interface morphology, the microstructure of the surface machined YSZ/Ti6Al4V brazing joint shows no significant difference with the non-machined one which has been characterised elsewhere^41^. Thus in this paper, the previously investigated microstructure of the joint which is YSZ/TiO + Ti_3_Cu_3_O/Ag(s,s)/TiCu_2_/TiCu/Ti_2_Cu/α + β Ti/Ti6Al4V is applied for further discussion. It can be seen that the surface machining process will not change the phase composition in the joint and mainly affect the interface morphology of the joint.

**Figure 3:**
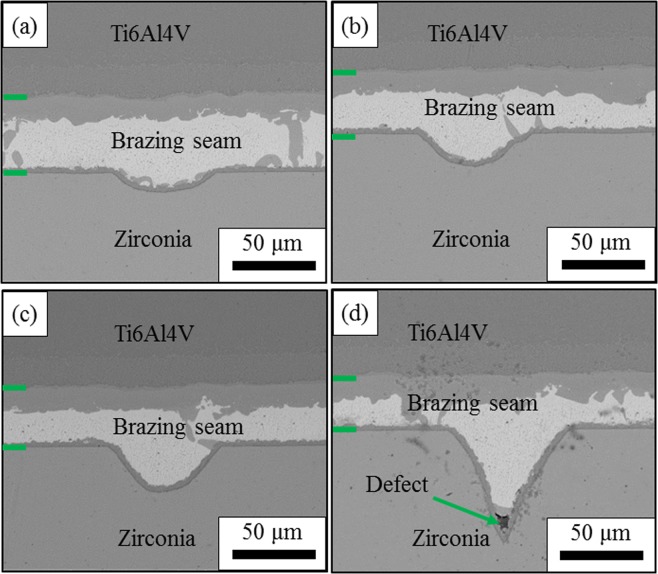
The effect of surface machining speed on the microstructure of the brazing joint between surface machined zirconia and T6Al4V alloy brazing joint. The figures show the microstructure of surface machined zirconia/brazing joint: (**a**) machining speed: 1000 μm/s, (**b**) machining speed: 600 μm/s, (**c**) machining speed: 200 μm/s, (**d**) machining speed: 100 μm/s, a defect can be observed at the bottom of machined grooves.

The microstructure of the joints achieved using the YSZ after surface machining with different machining speed as the substrates are shown in Fig. 3. The machining speed seems not to have a large influence on the microstructure of the joint despite the morphology of the interface between the YSZ and the brazing filler. As the machining speed decreases, the depth of the grooves increases. It can be seen that when the processing speed ranges from 200 μm/s to 1000 μm/s, the brazing filler can fill up the machined grooves and the defect-free joint can be achieved (Fig. 3(a–c)). However, when the machining speed declines to 100 μm/s, a sharp corner appears at the bottom of the groove and the brazing filler cannot reach the end of the corner, leaving a void at the interface, as shown in Fig. 3(d). This sharp defect could act as a source of stress concentration and might be detrimental to the reliability of the joint^42^. Thus it can be inferred that to achieve a defect free joint, the machining speed should be higher than 100 μm/s. It is also noted that the width of the brazing seam decreases with the increasing of the groove depth. This is because part of the brazing filler needs to fill up the grooves and less brazing filler is left in the brazing seam.

### Mechanical properties of the joint

**Figure 4:**
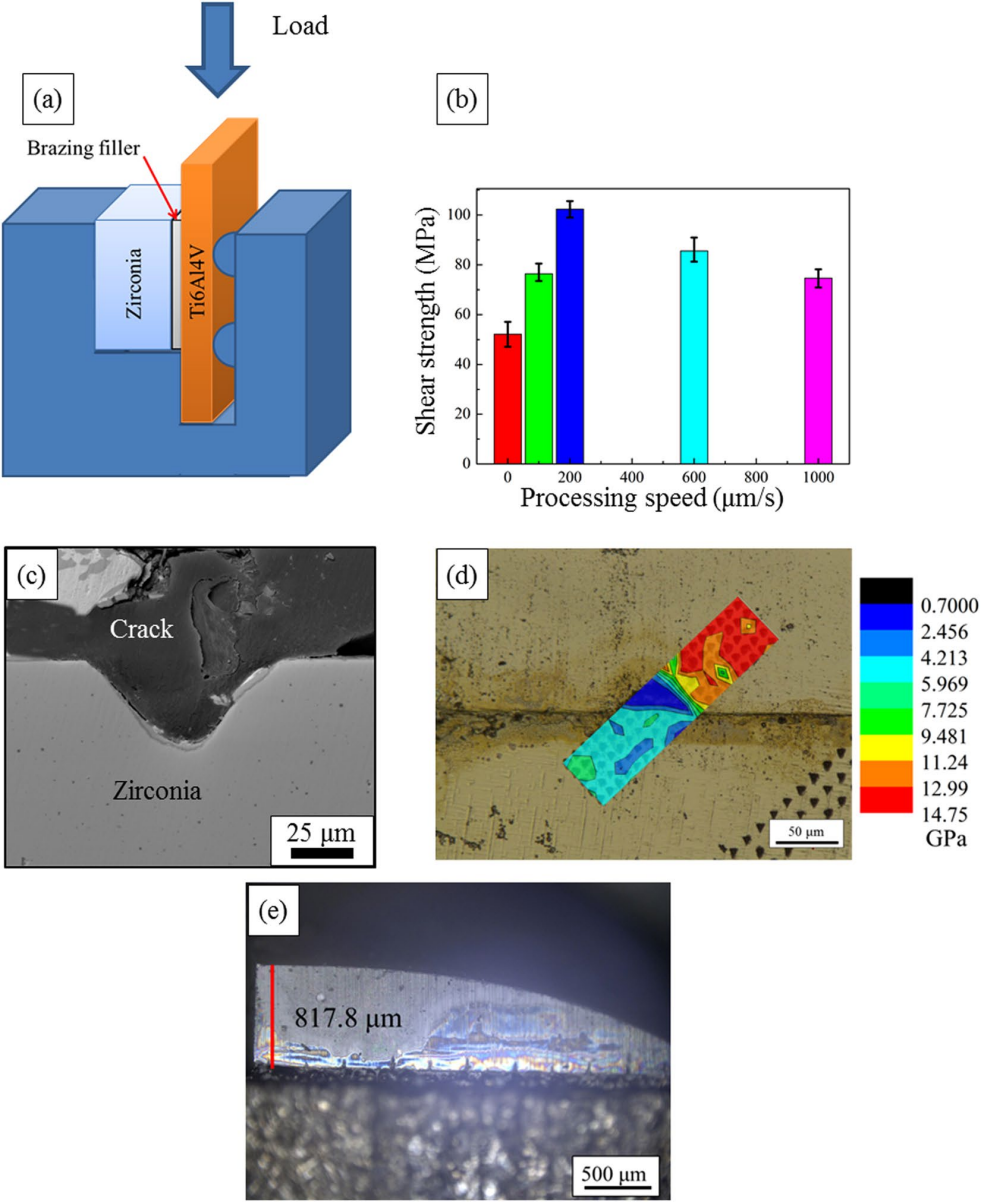
(**a**) The schematic of the shear test, showing the loading direction is perpendicular to the direction of the machined grooves, (**b**) the relationship between the shear strength and the processing speed, which first increases with the processing speed and then decreases, (**c**) the fracture morphology of the surface machined zirconia/Ti6Al4V brazing joint, (**d**) the hardness distribution across the interface in the joint, (**e**) the morphology of a failed surface micromachined YSZ/Ti6Al4V brazing joint.

The interface morphology has a close relationship with the residual stress distribution near the interface^30,37,43^, which is considered to have a significant influence on the strength of the joint. Thus shear testing was carried out to investigate the effect of the surface machining on the strength of the joint. The direction of load is perpendicular to the direction of the grooves as shown in Fig. 4(a). All the joints show brittle fractures and the load at fracture (*F*) is applied to calculate the strength of the joint (*τ*), which is achieved by formula (1).

1 $$\tau =\frac{F}{A}$$

where *A* is the area of the nominal joined surface, which is 4 mm × 4 mm = 16 mm^2^ in this research.

As shown in Fig. 4(b), when using the unmachined YSZ as the substrate, the shear strength of the joint is relatively low (~50 MPa). As machining speed increases, the shear strength of the joint first increases then decreases. The highest strength of about 103 MPa was achieved when the machining speed was 200 μm/s, which was 96.2% higher than that of the joint using the flat YSZ as the substrate. To investigate the fracture position of the joint, the cross section of the joint fracture with a rumpled interface was observed by SEM, as shown in Fig. 4(c). It can be seen that the crack leading to the failure of the joint mainly propagates inside the reaction layer between the YSZ substrate and the silver solid solution, indicating that the interface region is the “weak link” of the joint. This result is similar to that of the YSZ/Ti6Al4V joint with a flat interface, where the fracture also happens in the reaction layer between the ceramic and brazing filler^41^. The hardness distribution across the joint is shown in Fig. 4(d), it can be seen that the hardness of the YSZ is the highest, which reaches about 13.8 GPa. The hardness measured at the interface between the ceramic and the brazing filler and the interface between the Ti6Al4V and the brazing filler are around 3.13 GPa and 4.84 GPa, respectively. The brazing seam has the lowest hardness, which is about 1.26 GPa. Figure 4(e) shows the morphology of another failed sample and it can be seen that crack leading to failure was firstly generated and propagated along the interface, and it then was deflected from the interface and continue to propagate about 810 μm away from the interface. This phenomenon will be explained later.

### FE modelling results

**Figure 5:**
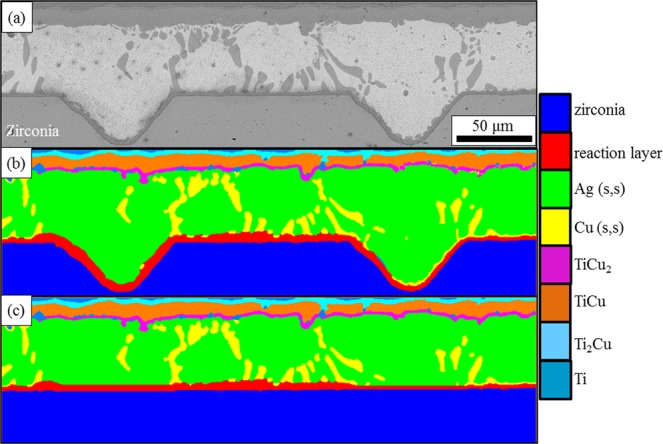
(**a**) The microstructure of surface machined zirconia/brazing joint when the machining speed is 200 μm/s, (**b**) the segmentation result of the image shown in Fig. 6(a), (**c**) the microstructure model with the rumpled interface removed, Colour Key: Zirconia – dark blue; Reaction layer – red; silver solid solution - green; copper solid solution – yellow; TiCu2 – pink; TiCu – brown; Ti2Cu - cyan; Ti – blue.

It can be seen that the surface machining could dramatically improve the shear strength of the joint but it has no large effect on the phase composition and the fracture position. The only feature it affects in the joint is the interface morphology which has a great influence on the residual stress distribution after brazing and stress distribution during the shear test near the interface region. Thus image based FE models are set up to study the effect of the surface machining on these two features. Figure 5(a) is an SEM image showing the microstructure of the surface machined YSZ/Ti6Al4V joint. Segmentation was then carried out for Fig. 5(a) and this process labels each phase in the joint. The result is shown in Fig. 5(b). To study the effect of the interface morphology on the residual stress distribution in the joint, we carefully remove the rumpling interface using the Lasso tool in Avizo9.0 and a flat interface with the same size is placed at the original position. The reaction layer with the same thickness in Fig. 5(b) is inserted at the interface between the YSZ and the brazing filler, the result of which is shown in Fig. 5(c). Then these two figures were imported to OOF2 for mesh generation and then the obtained FE model was imported into Abaqus CAE to be solved. About 720 thousand elements are generated in the models. The residual stress in the joint is generated from the CTE mismatch and before the brazing filler solidifies, both the ceramic and Ti6Al4V are free to expand and shrink. Thus the residual stress can be generated only after the solidification of the brazing filler and only one step, which is the cooling procedure from 780 °C to room temperature, was modelled for residual stress calculation. The mechanical properties of the various phases in the joint are listed in Table 1 and achieved either from references^37,44^ or calculated using Jmatpro software.

**Table 1:** Mechanical properties of various phases in the joint.

Phase	E (GPa)	CTE (°C^−1^)
YSZ	200	11 × 10^−6^
Ti6Al4V	116	8.5 × 10^−6^
Reaction layer	200	15.1 × 10^−6^
Ag	85	19 × 10^−6^
Cu	130	16.7 × 10^−6^
Ti_2_Cu	150	10.83 × 10^−6^
TiCu	170	16.64 × 10^−6^
TiCu_2_	162	18.91 × 10^−6^

### Residual stress distribution

**Figure 6:**
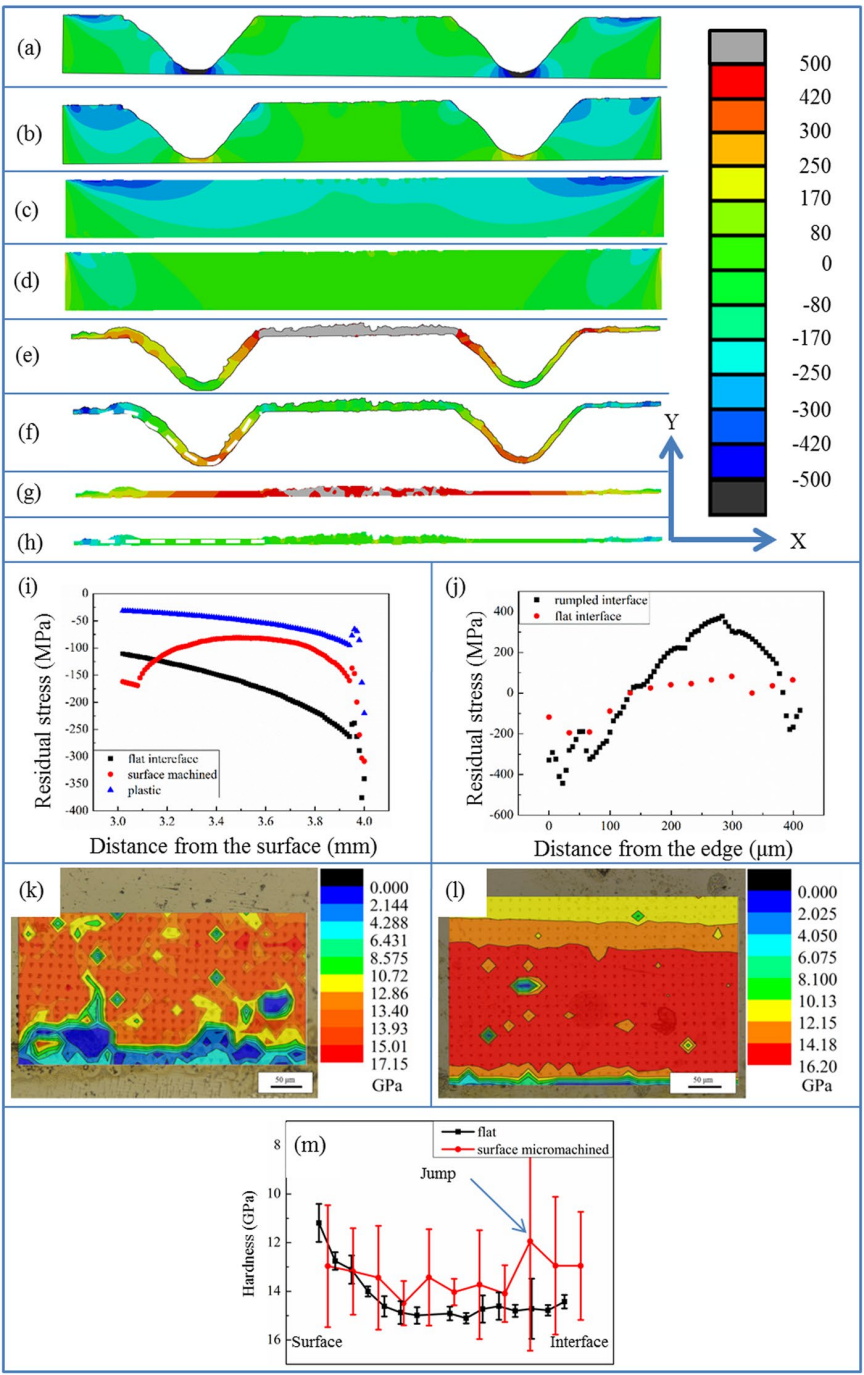
The modelled residual stress distribution in Zirconia and reaction layer by image based modelling, (**a**) the in plane residual stress in zirconia of the joint with a rumpled interface, (**b**) the out of plane residual stress in zirconia of the joint with a rumpled interface, (**c**) the in plane residual stress in zirconia of the joint with a flat interface, (**d**) the out of plane residual stress in zirconia of the joint with a flat interface, (**e**) the in plane residual stress in the reaction layer of the joint with a rumpled interface, (f) the out of plane residual stress in the reaction layer of the joint with a rumpled interface, (**g**) the in plane residual stress in the reaction layer of the joint with a flat interface, (**h**) the out of plane residual stress in the reaction layer of the joint with a flat interface. (**i**) The in plane residual stress distribution as a function of depth in zirconia with a rumpled interface and flat interface, **(j**) the residual stress distribution along the path drawn in figure (**f**,**h**). The hardness distribution in the interface region of (**k**) a surface micromachined YSZ/Ti6Al4V joint, regions with relatively low hardness can be found adjacent to the grooves in the YSZ substrate, (**l**) a flat YSZ/Ti6Al4V joint, the measured hardness value is decreasing from the interface to the surface of the YSZ, (m) The averaged hardness distribution as a function of depth in a surface micromachined YSZ/Ti6Al4V joint and a flat YSZ/Ti6Al4V joint, a “jump” feature can be observed in the surface micromachined YSZ/Ti6Al4V joint hardness trend.

In general, the failure of a layered structure such as the ceramic/metal joint and ceramic coatings consists of the following procedures: the generation of the cracks, the propagation and the connection of the generated cracks, and then the actual failure happens. These procedures are closely related to the residual stress distribution in the joint. Thus to understand the mechanism of improvement of the reliability of the YSZ/Ti6Al4V brazing joint by the surface machining on the YSZ substrate, the residual stress distribution in the joint was firstly investigated via FE modelling. The modelled residual stress distribution in the YSZ substrate and the reaction layer which is the failure position of the joint is shown in Fig. 6. Both the in plane residual stress (S11) and the out of plane residual stress (S22) are calculated. It can be seen that the in plane residual stress in both the surface machined (Fig. 6(a)) and the flat YSZ (Fig. 6(c)) is generally compressive and the distribution is symmetrical due to the geometry of the model. For the flat YSZ substrate, the residual stress is generally increasing as it approaches the interface while for the surface machined YSZ, compressive stress concentration regions can be observed at the concave of the rumpling interface. Since the in plane residual stress is compressive, it will not be the reason for the crack generation. To study the influence of surface machining on the overall residual stress distribution in the YSZ substrate, we calculate the overall residual stress distribution as a function of depth by averaging the stress values (using a python script) of each element within the distance ranges from the surface. Figure 6(i) shows a comparison of the in plane residual stress distribution between the surface machined YSZ and the flat one. It can be seen that the residual stress distribution near the flat interface is very similar to previous reports^11,32,34,35^, which increases from the surface to the interface in a uniform trend. However, the residual stress distribution near the rumpled interface shows significant differences. The residual stress is compressive, which first increases from the surface to the interface, then decreases and increases again to the interface, resulting in a “jump” feature in the residual stress trend. This phenomenon is similar to the residual stress distribution as a function of depth in thermal barrier coatings with a rumpled interface^20,37,45^. This residual stress trend indicates that despite a maximum residual stress value appears at the interface of the joint, there is another maximum residual stress value about 800–900 μm away from the interface. This phenomenon could be correlated to the morphology of the failed sample shown in Fig. 4(e).

From the previous reports and our observation, the directions of cracks leading to the failure of the brazing joint are usually parallel to the interface of the joint. Thus the out of plane residual stress instead of the in plane stress will act as the driving force for the initiation of the crack that leads to the joint’s failure. The out of plane residual stress distribution near the rumpled and the flat interface in the YSZ substrate is shown in Fig. 6(b,d). It can be seen that the out of plane residual stress near the flat interface is generally tensile and two stress concentration regions exist at the edges of YSZ near the interface. According to the previous report^46^, some brazing joint between ceramic and metal may fail directly after the cooling procedure, where the crack leading to failure initiates from the edge of the ceramic near the interface. This modelling result corresponds well with this observation. YSZ is a kind of ceramic with relatively high fracture toughness and thus this kind of failure mode was not observed in our experiment. For the models which take the rumpled interface into consideration (Fig. 6(b)), the out of plane residual stress distribution changes a lot. The compressive out of plane residual stress is generated besides the grooves and the tensile stress concentration regions at the two edges disappear, which could suppress the failure mode mentioned above. It needs to be noted that tensile out of plane residual stress can be found at the concave of the grooves. However, even though cracks can be generated by this tensile stress, the propagation of the cracks will be suppressed by the compressive stress besides the grooves.

**Figure 7:**
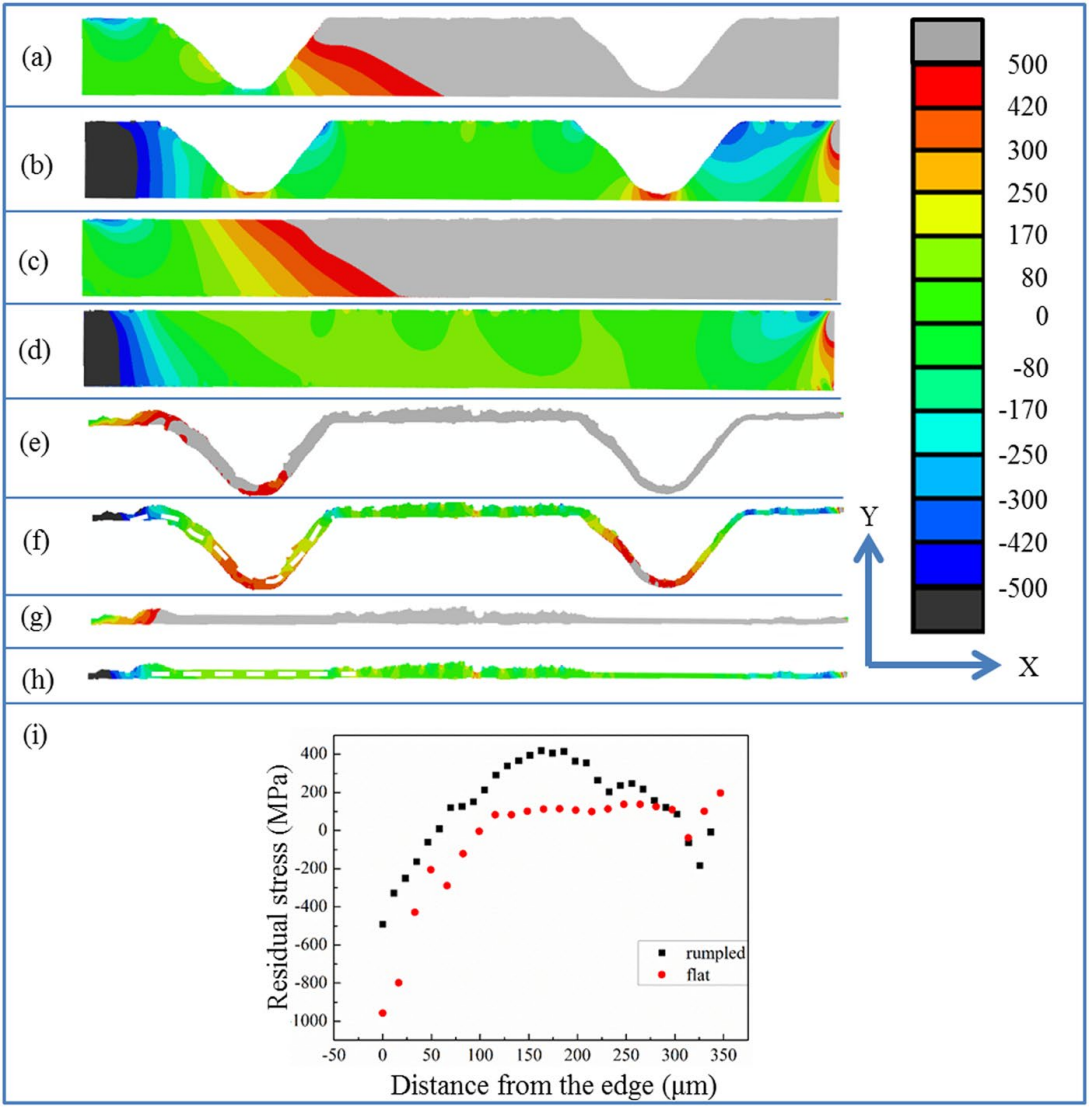
The modelled stress distribution in Zirconia and reaction layer by image based modelling upon shear testing, (**a)** the in plane stress in zirconia of the joint with a rumpled interface, (**b**) the out of plane stress in zirconia of the joint with a rumpled interface, (**c**) the in plane stress in zirconia of the joint with a flat interface, (**d**) the out of plane stress in zirconia of the joint with a flat interface, (**e**) the in plane stress in the reaction layer of the joint with a rumpled interface, (**f**) the out of plane stress in the reaction layer of the joint with a rumpled interface, (**g**) the in plane stress in the reaction layer of the joint with a flat interface, (**h**) the out of plane stress in the reaction layer of the joint with a flat interface, **(i**) the stress distribution along the path drawn in figure (**f**,**h**).

In our experiment, the failure happens inside the reaction layer between the YSZ and the brazing filler. Thus the residual stress distribution inside the reaction layer was modelled, the results of which are shown in Fig. 6(e–h). It can be seen that the in plane residual stress (Fig. 6(g)) for the reaction layer along the flat interface is tensile and the value of the tensile stress is larger in the middle of the interlayer than that at the edge. However, this tensile stress is unlikely to cause the failure of the joint since the crack induced by the in plane tensile stress is perpendicular to the interface and it will be stopped by the compressive in plane stress in YSZ. The in plane residual stress (Fig. 6(e)) in the reaction layer of the rumpled interface is also tensile and the magnitude of the tensile stress is higher in the middle than that at the edge as well. For similar reasons to that of the flat interface, the in plane tensile stress in the reaction layer along the rumpled interface should also not be the reason for the failure of the joint. Figure 6(h) shows the contour plot of the out of plane residual stress in the reaction layer along the flat interface and Fig. 6(j) shows the residual stress distribution along the path drawn in Fig. 6(h). The out of plane residual stress is generally tensile and compressive stress can only be found at the edge of the reaction layer. Once a crack parallel to the interface was generated, it will penetrate through the majority part of the reaction layer until the edge which makes the joint tends to fail upon a small external load. While for the rumpled interface joint, as shown in Fig. 6(f), the out of plane residual stress is tensile at the concave of the groove. From the residual stress trend (Fig. 6(j)) along the path drawn in Fig. 7(f), we can see that compressive stress regions exist at the boundary of the grooves. In this case, the cracks tend to initiate at the concave of the grooves, but compressive stress regions at the boundary of the grooves could act as an effective crack stopper. Thus compared to the joint with a flat interface, the cracks in the reaction layer of the joint with a rumpled interface is more unlikely to penetrate through the whole joint and cause the eventual failure. From the above discussion, we can see that the surface machining on the YSZ could remove the tensile stress concentration region in the ceramic and generate compressive out of plane residual stress in the reaction layer. These all contribute to the improvement of the joint strength.

The previous models all assume that the materials in the model are purely elastic. However, according to previous research, plastic deformation of the Ag(s, s) may happen during the cooling process. To investigate the effect of the plastic deformation on the residual stress distribution in the joint, we set the yielding strength of silver as 85 MPa and the modelled result is shown in Fig. 6(i). It can be seen that the value of the residual stress decreases when the plastic deformation of silver is taken into consideration in the model, but it has no large influence on the trend of the residual stress distribution. Thus, for simplicity, the elastic model is applied to predict the trend of the residual stress in this research. This result also indicates that the plastic deformation of soft materials such as silver in the joint could help to release the residual stress in the joint.

It has been reported that the compressive stress could lead to a higher measured hardness and tensile stress results in a lower measured hardness^47,48^. Thus, hardness mapping measurements are carried out in the interface region of the YSZ substrate for the joint with rumpled and flat interface to validate the simulation results. It can be seen from Fig. 6(k) that regions with relatively low hardness can be found adjacent to the grooves in the YSZ substrate, from which it can be inferred that the compressive stress in the region adjacent to the grooves is smaller than the region far away from the grooves. This result corresponds well with the simulated residual stress distribution, as shown in Fig. 6(a). The residual stress in the YSZ of a brazing joint with a flat interface is also compressive. In Fig. 6(i), we can observe that the measured hardness is highest near the interface and decreases from the interface to the surface. This result indicates that the compressive residual stress is increasing from surface to the interface, which also agrees with the modelled residual stress distribution shown in Fig. 6(c). To further validate the simulation results, the average hardness as a function of depth in both types of the joints is plotted, as shown in in Fig. 6(m). It can be seen that the a “jump” feature can be found in the average hardness trend in the surface micromachined YSZ/Ti6Al4V joint and the hardness in the flat YSZ/Ti6Al4V joint is increasing from the surface to the interface. These results could also help to prove that the modelled residual stress distributions are reliable.

### Shear test modelling

As discussed above, the strength of the joint can be improved by carrying out surface machining on YSZ before brazing it with metal and the actual strength of the joint was measured by shear testing. So one more step which is the process of the shear test is added to the model after the cooling procedure. A displacement of 0.02 mm to the X-positive direction is applied on the Ti6Al4V substrate while the displacement in the X direction of the YSZ right edge and its rotation around the Z axis are fixed as zero to simulate the shear testing process in our experiment. The predicted stress distribution in YSZ after surface machining is shown in Fig. 7. Upon shear testing, the in plane stress distribution in the YSZ substrates with both a rumpled interface (Fig. 7(a)) and a flat interface (Fig. 7(c)) is similar which becomes unsymmetrical. Tensile stress is generated near the interface on the side with the same direction of the displacement in YSZ while compressive stress is induced on the opposite side. These tensile regions could generate cracks. But in this research, probably due to the relatively high roughness of the YSZ substrate, this kind of failure mode was not observed.

While the out of plane stress (Fig. 7(d)) of the ceramic with flat interface upon shear test is tensile at the side to the X-positive direction, and tensile stress decreases from the X-positive side to the X-negative side. A tensile stress concentration region can be observed at the corner between the X-positive edge of the YSZ substrate and the interface, which could act as the initiation position of the cracks. According to the previous report, some brazing joints will fail with crack generated from the corner of the ceramic near the interface, which corresponds well with this modelling result. But the ceramic used in this experiment is YSZ, which has relatively high toughness and so the weak position of the joint shifts from the ceramic to the reaction layer. The out of plane stress distribution in the YSZ substrate upon shear testing of a surface machined YSZ/Ti6Al4V brazing joint is shown in Fig. 7(b). Positive out of plane stress can be found near the X-positive edge of YSZ, which is similar to that of the joint with a flat interface. However, compressive stress regions can be observed near the interface adjacent to the grooves, which could inhibit the cracks propagating in the interface region. Similar to the residual stress distribution, two regions with tensile stress can still be observed at the concave of the grooves. Again, this tensile stress is unlikely to be the reason for the failure of the joint since besides these regions with tensile stress, compressive stress is presented. Thus comparing to the joint with a flat interface, the cracks tend not to be generated and propagate in the ceramic of a joint with a rumpled interface.

The fracture of the joint was found to happen in the reaction layer. Thus to understand the mechanism for the improvement of the joint strength via surface machining on the YSZ substrate, the stress distribution in the reaction layer during the shear test is studied and the results are shown in Fig. 7(e–h). It can be seen that the in plane stress in the reaction layer along the flat interface is tensile upon shear testing (Fig. 7(g)). This tensile stress may generate some vertical cracks inside the reaction layer. However, because the phases adjacent to the reaction layer are silver and YSZ, these vertical cracks are unlikely to propagate and cause the actual failure. Similarly, the in plane stress in the reaction layer along the rumpled interface is also tensile and the value of the tensile stress is higher at the X-positive side, as shown in Fig. 7(e). For the same reason with that in the flat interface case, this tensile stress also tends not to cause the actual failure of the joint, even though small vertical cracks may be induced by this tensile stress.

As shown in Fig. 4(c), the crack causing the failure is parallel to the interface. So the out of plane stress in the reaction layer upon shear testing is more relevant to the failure of the joint. The out of plane stress distribution in the reaction layer along the flat interface during the shear test is depicted in Fig. 7(h). We can find that the out of plane stress in most part of the reaction layer is tensile. Cracks could be generated and easily propagate through majority part of the reaction layer. In this case, the crack leading to the failure of the joint will be along the interface, which corresponds well with the previous observations^41^. When the surface of the YSZ is machined by femtosecond laser, the out of plane stress in the reaction layer upon shear testing is greatly altered, which is shown in Fig. 7(f). Even though tensile stress can be found at the concave of the grooves and cracks could possibly be generated at these positions, compressive stress is presented beside the grooves, which could inhibit the propagation of the cracks, as shown in Fig. 7(i). This kind of stress distribution could also help to reduce the tendency for the cracks to connect with each other and cause the actual failure of the joint. Also, the cracks leading to the failure of the joint with a rumpled interface needs to keep changing its propagation directions, which inevitably increases the interfacial toughness between the ceramic and the brazing filler compared to the joint with a flat interface. This could also help to improve the strength of the joint.

**Figure 8:**
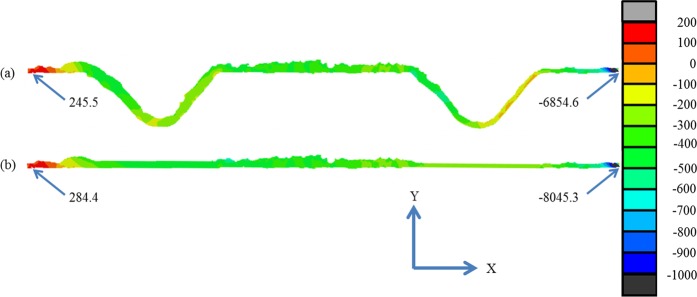
The predicted shear stress distribution in the reaction layer by FE models upon shear test, (**a**) rumpled interface, (**b**) flat interface.

Even though ceramics are more resistant to shear stress than tensile stress^49^, upon shear testing, during which the value of shear stress keeps increasing, the failure could also be possibly generated by the shear stress. So the shear stress inside the reaction layer (where the failure happens) during the shear testing is predicted using the FE method. The result is shown in Fig. 8. It can be seen that in both cases, the maximum shear stress is observed at the two ends of the reaction layer. The maximum shear stress value in the reaction layer of the joint with a rumpled interface (Fig. 8(a)) is lower than that of the joint with a flat interface (Fig. 8(b)). So the cracks induced by the shear stress are more unlikely to be generated and propagate in the reaction layer with a rumpled interface.

## Methods

### Sample preparation and brazing procedure

The ceramic used in this study was 3 mol% YSZ with the size of 4 mm × 4 mm × 4 mm, and the Ti6Al4V alloy was machined into 10 mm × 6 mm × 3 mm pieces before the brazing procedure. The composition of the brazing filler is Ag-28Cu (wt%) and it was sandwiched between the ceramic and the metal. The thickness of the brazing filler was 50 μm and the size of which was 4 mm × 4 mm to fit the ceramic. The brazing process was carried out in a vacuum furnace under a vacuum lower than 2 × 10^−3^ Pa at 870° for 5 min. The cooling rate was set as 10 °C/min to inhibit the possible cracking.

### Surface machining

The surface machining of the YSZ was carried out in an air atmosphere using a Yb:KGW laser system (Pharos-15W, Light Conversion), emitting at a wavelength of 1030 nm. The pulse energy was 200 μJ and the repetition rate was set as 1 kHz. The sample was mounted on a computer driven stage which could move in X and Y directions and help pinpoint the laser spot to the desired position on the surface of YSZ. The schematic depicting the geometry of the surface machining is shown in Fig. 1(a). During machining, the sample will be moved horizontally to make the laser scan through the sample surface line by line. The distance between each machined groove is 200 μm.

### Microstructure characterisation

After brazing, the samples were mounted in resin, cross sectioned by the diamond cutting wheel, ground and polished to 1 μm finish. Before observation, the surface of the sample was coated by ~20 nm thick gold coating. The morphology of the machined YSZ surface and the microstructure of the joint were observed by SEM (Quanta 200) coupled with an EDS detector.

### Image based FE modelling

The achieved microstructure of the joint by SEM was saved as tiff files, and then these images were segmented using the trainable Weka segmentation plugin^50^ in the Fiji software^51^ and Avizo Lite 9.0 software (FEI Visualisation Science Group). Then the segmented images were imported into OOF2^52^ to generate a mesh for FE model calculation. The meshing was carried out in an adaptive mesh generation approach which could adjust the element size depending on the microstructural features (mainly interface). The mesh was then exported as INP files and imported into an FE solver (Abaqus 6.13) to calculate the residual stress distribution. To investigate the effect of the surface machining on the residual stress distribution in the YSZ/Ti6Al4V joint, the uneven interface in the original segmented image was smeared out using the lasso tool in Avizo 9.0, and a flat interface which has a reaction layer with the same thickness was placed at the original position. Then this image was exported, meshed and the obtained model was solved. The predicted stress distribution was compared with that achieved using the actual microstructure to determine the effect of the surface machining on the stress distribution in a YSZ/Ti6Al4V joint.

### Mechanical test

The strength of the joint was evaluated by shear testing, which was carried out on a universal mechanical testing machine. The loading speed was 0.5 mm/min and for each test, at least 5 samples were used. The hardness evolution through the interfaces was measured using nanoindentation. The hardness in a matrix of 5 × 20 across the interface was measured and since the width of the brazing seam is small (<100 μm), to improve the resolution of the measurement, the matrix had an angle of 45° to the interface counterclockwise. The penetration depth of the indenter was fixed at 1000 nm and the distance between each indenter was 15 μm.

## Conclusions

In this paper, a novel method to improve the bonding between YSZ and Ti6Al4V alloy is developed by surface micromachining on the YSZ substrate via femtosecond laser, the effect of the processing speed on the microstructure and mechanical property of the joint is investigated, the influence of the surface machining on the residual stress distribution after brazing and the stress distribution in the joint upon shear testing was predicted using image based modelling. Several conclusions have been drawn from this study:

1. Grooves can be successfully manufactured on the surface of the YSZ substrate using a femtosecond laser. The processing speed has no large effect on the width of the machined grooves, but with the decreasing of the processing speed, the depth of the machined grooves increase. The brazing alloy could fill up the grooves when the machining speed is no less than 200 μm/s and when the processing speed is lowered to 100 μm/s, defects can be observed at the sharp corner of grooves. Despite the interfacial morphology, the surface machining is found not to affect the microstructure of the joint and shear strength of the joint first increases with the processing speed and then decreases. The highest joining strength of 103 MPa is achieved when the processing speed is 200 μm/s which is about 95.2% higher than that using the flat YSZ as the substrate.
2. According to image based modelling, the in plane residual stress in both the surface machined and flat YSZ substrate are compressive. The residual stress in the flat ceramic is increasing from surface to the interface in a uniform trend, while the residual stress in the surface machined first increases from the surface to the interface, decreases a little and then increases again to the interface. This results in a “jump” feature in the residual stress trend. Out of plane tensile residual stress concentration regions can be observed at the corner of the ceramic near the interface, while the surface machining could eliminate these tensile stress concentration regions. The in plane residual stress in the reaction layer for both the flat and the rumpled interface joint are tensile. The out of plane residual stress in the flat reaction layer is tensile and for the rumpled reaction layer, the residual stress at the concave is tensile while the residual stress becomes compressive at the boundary of the grooves.
3. Upon shear testing, the in plane stress in the YSZ substrate for both the flat and the rumpled interface become unsymmetrical. The tensile out of plane stress in the YSZ can be found in both cases. For the joint with a rumpled interface, compressive stress is presented beside the region with tensile stress. The in plane stress in the reaction layer is tensile during shear testing for both the flat and the rumpled interface. The out of plane stress in the reaction layer is tensile in the joint with a flat interface which could drive the propagation of the cracks. The stress in the reaction layer at the concave of the rumpled interface during shear testing is tensile which may be the driving force for the generation for the cracks, but the propagation of the cracks will be inhibited by the compressive out of plane stress at the boundary of the grooves. The maximum shear stress in the reaction layer can be deceased by the surface machining of the YSZ substrate, which also increases the reliability of the joint.
